# The Supply of Doctors

**Published:** 1917-05-26

**Authors:** 


### THE SUPPLY OF DOCTORS.

As our pages have for some weeks borne testimony, the country is in process of being swept bare of doctors of military age: in a few weeks at the outside there will be no such thing as a practising civilian doctor under forty-one, except the merest handful of absolutely indispensable surgeons on the staffs of the largest hospitals. This sweep up, together with the prospect of the arrival of 1,000 doctors from America, should completely solve any difficulties the authorities may have apprehended in respect of medical officers for our armies. The advance guard of the American contingent (twenty doctors and a large number of nurses and orderlies) has already arrived; and the full thousand, it is generally understood, will be available reasonably soon. In the opinion of many from the Fronts, it is still true that better organisation and distribution of the medical officers already in the Army is both feasible and overdue; and it is very much to be desired that the Army Medical Service authorities shall apply their talents to this doubtless difficult but far too much neglected problem. The sacrifices made by the older men of military age, those over thirty let us say, are grievous enough, without the added reflection that wiser counsels at the War Office might have rendered them unnecessary. Rightly or wrongly, that is what some of the men at the Front do feel, for we have had it direct from their lips.

Meanwhile, there are aspects of the supply of doctors for the civilian population at home which are encouraging, as well as some that are less so. Those medical men whom years or medical unfitness prohibit from joining the R.A.M.C. are mostly working double tides: incidentally, their practices, for the most part, are appreciating rapidly in receipts, and therefore also in goodwill. There is a general disposition, marred here and there by exceptions, to "play the game" by the absent doctors, and to refrain from poaching the latter's practices. Still, even with the best will in the world, it is inevitable that those at home should profit at the expense of those abroad, and there is no way of avoiding this. It is said, apparently with truth, that there are young "conscientious objectors" in the medical profession whose alleged scruples against healing the wounded do not prevent them from settling in practice where other men have left gaps and from gathering up all the patients they can. One can believe anything of a "conscientious objector"; but it will be no credit to the British Medical Association and the licensing bodies if they fail to find a way of preventing this sort of conduct, or of penalising it so heavily as to render it unattractive. The State, greatly to its own detriment, in our opinion, and in deference to political prejudices of a type that the war has almost swept away, has recognised the "conscientious objector" and given him certain rights. We still hope, after the war, to see a revision of his position, and a withdrawal of all civic rights whatsoever, including the vote and the capacity to hold any public office of any kind. Meanwhile unofficial corporations can do much to express that disapproval which is deeply ingrained in the public mind of the conduct of those who profess the anti-social doctrines of "conscientious objection" to serve the State in war. On the whole, the outlook is bright from the point of view of the State, though it entails individual hardships, as war does for many other sections of the population.

